# Effects of Exposure to Extreme Artificial Light at Night on Liver Oxidative Damage and Gut Microbiota During Pregnancy and Lactation in Mice

**DOI:** 10.3390/ani16081171

**Published:** 2026-04-11

**Authors:** Ting Huang, Wenting Li, Xinyuan Dong, Wenjing Li, Mengmeng Jiang, Junhe Wang, Jing Wen

**Affiliations:** 1 School of Life and Environmental Sciences, Wenzhou University, Wenzhou 325035, China; huangting@mail.ustc.edu.cn (T.H.); lwt@stu.wzu.edu.cn (W.L.); dongxinyuan@stu.wzu.edu.cn (X.D.); 23211231128@stu.wzu.edu.cn (W.L.); 25451043010@stu.wzu.edu.cn (M.J.); 25211331232@stu.wzu.edu.cn (J.W.); 2 Division of Life Sciences and Medicine, University of Science and Technology of China, Hefei 230026, China; 3 Key Laboratory for Water Environment and Marine Biological Resources Protection in Zhejiang Province, Wenzhou 325035, China

**Keywords:** light pollution, photoperiod, mouse immune system, antioxidant capacity, passage effect

## Abstract

Chronic exposure to artificial light at night (ALAN) is increasingly recognized as an environmental risk factor that disrupts circadian regulation of endocrine and metabolic systems. In this study, we investigated the effects of extreme ALAN on oxidative stress and gut microbiota composition in mice using two complementary experiments. In Experiment 1, adult female mice were maintained under either as a standard 12 h light/12 h dark cycle (12 h group) or continuous 24 h light exposure (24 h group) throughout pregnancy and lactation. In Experiment 2, the offspring from the 12 h group were maintained under the same photoperiod, whereas offspring from the 24 h group were divided into a 12 h light/12 h dark group or a continuous 24 h light group, with treatments initiated on postnatal day 19 and continued until 2 months of age. For all 12 h groups, light exposure occurred from 8:00 to 20:00. Compared with dams in the 12 h group, dams exposed to continuous light exhibited significantly increased catalase activity, while their offspring maintained under the 12 h photoperiod showed elevated glutathione levels. No significant changes were detected in immune organ indices. These results suggest that extreme ALAN modulates antioxidant defenses, potentially reflecting adaptive responses to oxidative stress. Moreover, offspring exposed early to extreme ALAN showed significantly reduced gut microbial α-diversity, accompanied by decreased abundances of *Firmicutes*, *Bacteroidota*, *Campylobacterota*, and *Desulfobacterota*, and an increase in *Proteobacteria*. Notably, *Verrucomicrobiota* and *Akkermansia* failed to recover following photoperiod normalization, indicating persistent microbiota dysbiosis. Overall, these findings demonstrate that extreme ALAN induces oxidative stress and long-lasting alterations in gut microbiota composition, highlighting potential health risks associated with night-time light pollution.

## Simple Summary

In order to explore the effect of extreme artificial light at night (ALAN) on oxidative stress and gut microbiota composition in mice, we examined the antioxidant indices and gut microbiota in maternal mice and their adult offspring under nocturnal light exposure. In maternal mice, the effects of short-term exposure appeared to be partially counterbalanced by antioxidant regulation and limited microbial changes. In contrast, offspring subjected to extreme ALAN exhibited persistent alterations in oxidative markers and gut microbiota composition that were not reversed under the experimental photoperiod recovery conditions tested. These findings demonstrate that extreme ALAN induces oxidative stress and long-lasting alterations in gut microbiota composition, highlighting potential health risks associated with night-time light pollution.

## 1. Introduction

Artificial light at night (ALAN) is a pervasive form of light pollution that can disrupt natural light–dark cycles and adversely affect mammalian behavior, reproduction, and physiological processes, including metabolism, oxidative damage, immune responses, and neurological function [1,2,3]. The intensity, wavelength, and exposure duration of light are important factors mediating these effects [4]. Previous studies have shown that higher light intensities (50 Lux) and correlated color temperature (CCT) suppress cage activity in house mice [5]. Chronic exposure to artificial dim blue light (approximately 5 Lux) significantly promotes hepatic lipid accumulation in obese or diabetic mice [6]. Mice exposed to a long photoperiod (>16 h light/day) also exhibit significantly increased body weight during the postnatal period [7]. Additionally, accumulating evidence indicates that excess ALAN increases the risk of metabolic and mood disorders, such as type 2 diabetes and depression, in the diurnal sand rat *Psammomys obesus* [8,9]. Such exposures can directly or indirectly impair immune function and induce oxidative stress, primarily through disruption of circadian rhythms [10,11]. For example, constant light exposure has been shown to suppress immune function in crickets (*Teleogryllus commodus*), great tit nestlings (*Parus major*), BALB/c mice, and Siberian hamsters (*Phodopus sungorus*) [12].

Prolonged light exposure promotes excessive production of reactive oxygen species (ROS), leading to oxidative stress [13]. ROS can damage lipids, proteins, and DNA, thereby disrupting cellular redox homeostasis [14,15]. Consistent with this, light pollution significantly elevates ROS levels in the mouse brain [16]. Malondialdehyde (MDA), a key end product of lipid peroxidation, is widely used as a biomarker of oxidative damage, and its levels increase markedly in hepatocytes under oxidative stress [17]. To mitigate oxidative injury, cells rely on endogenous antioxidant defenses, including enzymes such as superoxide dismutase (SOD), catalase (CAT), and glutathione peroxidase (GSH-Px), as well as reduced glutathione (GSH) [18]. Increased antioxidant enzyme activity is typically a compensatory response to oxidative stress, with SOD catalyzing the dismutation of superoxide radicals into oxygen and hydrogen peroxide [19]. Prolonged light exposure has been reported to induce phosphorylation of tau protein, endoplasmic reticulum stress, synaptic loss, and altered antioxidant enzyme activity in rodents [20]. Oxidative stress also serves as an important upstream trigger of inflammatory responses, as persistent ROS accumulation activates inflammation-related signaling pathways and acute-phase reactions [21]. C-reactive protein (CRP) is a nonspecific acute-phase protein, and its levels rise sharply during infection or tissue injury, reflecting systemic inflammatory and immune homeostasis [22,23]. Glutamic-pyruvic transaminase (GPT) and glutamic-oxal(o)acetic transaminase (GOT) activities are widely used indicators of hepatic injury associated with oxidative stress, serving as sensitive markers of physiological stress and immune-related alterations [24].

In mammals, the diurnal oscillation of the gut microbiota is closely synchronized with host circadian rhythms [25]. Nocturnal light pollution has been shown to significantly alter the taxonomic composition, functional capacity, and community structure of the mouse gut microbiome [25]. Notably, probiotics can counteract oxidative stress by inducing anti-oxidative pathways, either by producing antioxidant enzymes such as SOD and catalase or by generating antioxidant metabolites, including folate and GSH [26,27,28]. Beneficial Firmicutes taxa, (e.g., *Lactobacillus*, *Bifidobacterium*, and *Butyricicoccus*) exert protective and anti-inflammatory effects on the intestinal epithelium [29]. In contrast, circadian disruption-associated dysbiosis is often characterized by enrichment of pro-inflammatory or pathogenic bacteria, which may further exacerbate oxidative stress [30]. Moreover, *Desulfovibrio vulgaris*, an opportunistic bacterium, has been linked to elevated ROS levels and reduced antioxidant capacity across multiple organs [31], whereas the abundance of *Butyricicoccus pullicaecorum* and *Faecalibacterium prausnitzii* is negatively correlated with the inflammatory response [32,33]. However, the mechanistic links among light exposure, oxidative stress, and gut microbiota dysregulation remain poorly understood.

Pregnancy and lactation are critical periods for fetal organogenesis and immune system maturation, during which maternal environmental exposures can shape offspring health via placental transfer and breast milk [34,35]. A wide range of maternal insults, including nutritional imbalance, endocrine disruption, pharmacological exposure, and circadian misalignment, have been shown to program adverse offspring outcomes, such as neurological deficits, immune dysfunction, hypertension, and hepatic steatosis [36]. ALAN applied during pregnancy may act as an endocrine disruptor, interfering with offspring development and circadian organization [37]. Continuous light exposure disrupts central and peripheral reproductive clocks, alters uterine physiology, and reduces pregnancy success in mice [38], while maternal chronodisruption impairs the establishment of the fetal circadian system [39]. Parent-offspring interactions involving gene regulation, immune cell development, and gut microbiota transmission are critical determinants of offspring immune maturation [40]. Nevertheless, most studies on light pollution have focused on adult individual-level outcomes, largely neglecting transgenerational effects and early exposure effects. Emerging evidence indicates that maternal circadian disruption exacerbates susceptibility to inflammatory diseases in the offspring of both sexes [41], suggesting that light pollution has lasting intergenerational consequences [42]. We therefore proposed a hypothesis that exposure to nocturnal artificial light during pregnancy and lactation may exert long-term effects on the offspring.

C57BL/6J mice are nocturnal animals with robust circadian rhythms and strong maternal care, resulting in high offspring survival rates, making them well suited for transgenerational studies. Research has revealed that in C57BL/6J mice, exposure to dim blue light at night significantly increases the levels of malondialdehyde (MDA, an oxidative stress product) in the hippocampus but decreases the levels of antioxidant enzymes glutathione peroxidase (GSH-Px), superoxide dismutase (SOD), and total antioxidants (T-AOC) in the hippocampus [43]. However, the transgenerational effects of light exposure on oxidative damage, antioxidant capacity, and metabolic outcome remain insufficiently explored. In this study, we assessed oxidative damage (MDA), antioxidant capacity (SOD, CAT, GSH), inflammatory marker (CRP) levels, and gut microbiota composition to evaluate the effects of extreme ALAN. Our objective was to investigate the intergenerational impact of nocturnal artificial light during pregnancy and lactation on hepatic oxidative stress and gut microbiota in mice.

## 2. Materials and Methods

### 2.1. Experimental Animals and Grouping

C57BL/6J mice were obtained from the laboratory breeding stock of Wenzhou University. Eighteen healthy 3-month-old female mice were taken and individually housed in plastic cages (29 cm × 18 cm × 16 cm) with wood-shaving bedding under controlled environmental conditions (23 ± 1 °C) and a standard 12 h light/12 h dark photoperiod (light on from 8:00 to 20:00; the light intensity at 100 cm above the floor was 150–200 Lux with a CCT of 3000–4000 K, and the light intensity inside the cage was 25–35 Lux. The animals had ad libitum access to food (Rat and Mouse Maintenance Diet, Beijing Ke’ao Feeding Co., Beijing, China; calorific value of 17.6 kJ/g) and water. In Experiment 1, after one week of acclimation, females were paired with healthy adult males for mating (5 days, 12 L:12 D, 8:00–20:00 light), and following mating, these females were rehoused individually and randomly assigned to one of two light-exposure groups: a 12 h light group (12 L:12 D, 8:00–20:00 light, *n* = 9) or a constant-light group (24 L, 0:00–24:00 light, *n* = 9). Light treatments were maintained throughout pregnancy and lactation (about 40 days), with the day of birth designated as lactation day 1. On postpartum day 20 (during 11:00–13:00), dams were euthanized by cervical dislocation, and tissue samples were collected. Experiment 2: a total of 24 male offspring derived from these dams were selected and allocated to three post-weaning light-exposure groups (*n* = 8 per group). Male offspring were selected from different litters as evenly as possible to avoid over-representation of a single dam. The animals assigned to three groups: (1) the 12 h light group (12 L:12 D), which was maintained under the standard photoperiod; (2) the 24–12 h group, in which offspring from the 24 h maternal group in Experiment 1 were housed with their dams under continuous light (24 L:0 D) before weaning and then directly transferred to a 12 L:12 D photoperiod on postnatal day 19, after which they were housed individually; and (3) the 24 h light group, which remained under continuous light (24 L:0 D). All offspring were housed with their dams before weaning. After weaning, they were housed individually under their respective lighting conditions until 8 weeks of age, at which time they were euthanized and sampled (during 11:00–13:00), with food and water provided ad libitum throughout the experiment. All experimental procedures complied with the guidelines of the Animal Care and Use Committee of Wenzhou University (WZU-2022-025).

### 2.2. Measurement of Body Weight and Food Intake

After the birth of the offspring, the body weights of the mother and offspring, as well as food weight, were measured at 17:00 for 18 consecutive days using an electronic balance (BT25S, Sartorius, Göttingen, Germany) with an accuracy of 0.1 g. The number of offspring was recorded daily, and any dead animals were recorded and disposed of in a timely manner. Food intake was calculated by subtracting the weight of the remaining feed blocks and feed residues in the rearing cage on the following day from the weight of the feed blocks measured on the previous day.

### 2.3. Metabolic Rate Measurement

Metabolic rate measurements in both female mice and male offspring were carried out using an open metabolometer (TSE, Baden-Württemberg, Germany). Daily metabolic rate (DMR) was measured in females on day 18 of lactation, whereas DMR measurements in male offspring were performed at 2 months of age. Gas was pumped into the respiratory chamber at a flow rate of 1 L/min, and the gas was dried and sampled through an oxygen analyzer (flow rate 0.38 L/min). Data were collected every 10 min, and metabolic rate was expressed as the rate of oxygen consumption per unit body weight (mL/h). Resting metabolic rate (RMR) was measured at 30 ± 1 °C for 3 h, during which time the animals were not allowed to drink or eat. DMR was measured at 23.0 °C ± 1 °C for more than 24 h. The animals were allowed to feed freely during the measurement. During the measurement period, the animals were allowed free access to food and water, with carrots provided as a source of both energy and hydration. The final DMR was expressed as the continuous oxygen consumption rate of mice for 24 h and was corrected for standard atmospheric pressure.

### 2.4. Determination of Calorific Value, Energy Intake, and Digestibility of Fecal Pellets

Bedding and fecal pellets were collected from days 13 to 15 of the lactation period, dried in a constant-temperature drying oven (60 °C) until a constant weight was reached, and fecal pellets were separated from the bedding and weighed (accurate to 0.001 g). Approximately 0.5–1.0 g of fecal pellets was then used for calorific value determination using an oxygen-nitrogen calorimeter (IKA C2000, Staufen, Germany). Before use, the calorimeter was calibrated using benzoic acid with a standard calorific value. The power supply of the oxygen-nitrogen calorimeter and the valve of the oxygen cylinder were then switched on sequentially. When the temperature of the instrument decreased to 20 °C, the start-up procedure was initiated, followed by a self-checking process, after which the instrument entered the standby measurement mode. The calculation formulas for gross energy intake (GEI), fecal energy (GEF), digestible energy intake (DEI), and digestibility are as follows [44,45]:

GEI (kJ/d) = intake (g/d) × feed dry matter content (%) × food energy value (kJ/g);

GEF (kJ/d) = dry weight of fecal pellets (g/d) × energy value of fecal pellets (kJ/g);

DEI (kJ/d) = GEI − GEF;

Digestibility (%) = (DEI/GEI) × 100%

### 2.5. Immunity Index

After lactating female mice and male offspring were euthanized by cervical dislocation following blood sampling, the thymus and spleen were rapidly excised, cleared of surrounding connective tissue, and weighed (to the nearest 1 mg). After weighing, the organs were put into 1.5 mL centrifuge tubes and quickly frozen in liquid nitrogen and then transferred to an ultra-low temperature-refrigerator (−80 °C) for storage. The immune organ index was calculated for the immune organs (thymus and spleen) with the following formula:

Immune organ index (IOI, mg/g) = weight of thymus or spleen (mg)/body weight (g).

### 2.6. Tissue and Organ Wet Weight

After euthanasia of lactating females and adult male offspring, tissues and organs, including liver, hindlimb skeletal muscle, and brown adipose tissue (BAT), were quickly excised. The heart, lungs, spleen, kidneys, large intestines, small intestines, stomach, and cecum (with contents removed) were also separated. In addition, abdominal fat, mesenteric fat, and testicular peritoneal fat were collected. All tissues and organs, as well as carcasses with viscera removed, were weighed separately (except for carcasses) and immediately frozen in liquid nitrogen. The frozen samples were then transferred to a −80 °C refrigerator for long-term storage.

### 2.7. Determination of Antioxidant Function and CRP (C-Reactive Protein)

Hepatic antioxidant status and liver function were evaluated using the following biochemical indices: liver total superoxide dismutase (SOD), catalase (CAT), malondialdehyde (MDA), glutathione peroxidase (GSH-Px), reduced glutathione (GSH), glutamate-pyruvate transaminase (GPT), and glutamic-oxal(o)acetic transaminase (GOT). At the time of sample collection, a portion of the liver was excised, placed in a sterile tube, and stored at −80 °C for antioxidant status and CRP determination. During the experiment, the frozen liver tissue was removed from the −80 °C refrigerator and placed on ice. The liver was accurately weighed to 0.1 g, and 1 mL of the corresponding extraction solution was added to it according to the manufacturer’s instructions. The tissue was then thoroughly homogenized on ice, followed by centrifugation at 138,000 *g*/min and 4 °C for 10 min (Eppendorf, Hamburg, Germany). The supernatant was transferred to a new 1.5 mL centrifuge tube, stored at 4 °C, and analyzed within 24 h. The activities of SOD, CAT, GSH-Px, GSH and the concentration of MDA were measured using the corresponding commercial assay kits. All indicators were quantitatively detected by colorimetry using a microplate reader (Thermo Scientific, Waltham, MA, USA). The target substances (enzymes or metabolites) in the samples underwent specific enzymatic or chemical reactions with the kit-matched reagents to generate colored products with characteristic light absorption. Blank and control wells were set, the absorbance values were measured at the corresponding wavelengths, and the absorbance differences (ΔA) were further calculated. Based on the kit-specific reaction principles, the results were calculated using the manufacturer-provided formulas to achieve quantitative analysis of the target indicators (Jiangsu Addison Biotechnology Co., Ltd., Yancheng, China). Specifically, the optical density (OD) values were measured at 450 nm for SOD, 510 nm for CAT, 412 nm for GSH-Px, 412 nm for GSH, and at 532 nm and 600 nm for MDA. The amplitude of absorbance change showed a good linear relationship with the content or enzyme activity of the target indicators in the samples, which laid a foundation for the accuracy of the quantitative results. Total protein concentration and C-reactive protein (CRP) concentration in the liver extract were determined using a BCA protein assay kit (Jiangsu Addison Biotechnology Co., Ltd., Yancheng, China) and a C-reactive protein assay kit (Nanjing BYabscience Technology Co., Ltd., Nanjing, China), respectively. Liver CRP concentrations were presented as μg per mg total protein. The OD values were measured at 562 nm for total protein and 450 nm for CRP. All experimental procedures were carried out according to the manufacturer’s instructions.

### 2.8. Determination of Gut Microbiota

At the time of animal sampling, the cecal contents were aseptically collected from the cecum, placed in 1.5 mL centrifuge tubes, and immediately frozen in liquid nitrogen, followed by storage at 80 °C. The samples were transported on dry ice to Meggie Biologicals for DNA extraction and 16S rDNA gene sequencing of the cecal contents [46]. Paired-end raw sequencing reads were quality filtered using fastp (https://github.com/OpenGene/fastp, version 0.19.6, accessed on 4 August 2023) and merged using FLASH (https://ccb.jhu.edu/software/FLASH/, version 1.2.11, accessed on 4 August 2023). The quality-controlled merged sequences were clustered into operational taxonomic units (OTUs) at 97% similarity, and chimeric sequences were removed using UPARSE v7.1 (http://drive5.com/uparse/, 4 August 2023). OTU taxonomic annotation was performed using the RDP classifier (http://rdp.cme.msu.edu/, version 2.11) against the 16S rRNA gene database (v138) with a confidence threshold of 70%, and microbial community was quantified at different taxonomic levels. Functional prediction of microbial communities was conducted using PICRUSt2 (version 2.2.0).

### 2.9. Tissue State-4 Respiration and COX Determination

Mitochondrial state IV respiration (S4R) and cytochrome c oxidase (COX) activity in liver, hindlimb skeletal muscle, and brown adipose tissue (BAT) from female mice and male offspring were determined using a platinum oxygen electrode dissolved oxygen meter (Model DW-1, Hansa Technology, Norfolk, UK). Both S4R and COX were measured at 30 °C in a 2 mL reaction mixture. Approximately 0.1 g of tissue (liver, muscle, and BAT) was accurately weighed using an electronic balance and homogenized with 0.9 mL of the extraction solution (mass ratio 1:9). Tissue homogenates were prepared by thorough grinding using a high-throughput tissue milling instrument (Ningbo Xinzhi Biotechnology Co., Ltd., Ningbo, China). The homogenates were kept in an ice-water bath, and all assays were completed within 24 h.

For the determination of mitochondrial state-4-respiration, 0.1 mL of tissue homogenate was combined with 1.9 mL of S4R reaction solution (0.225 mol/L sucrose, 5 mmol/L Tris-HCl, 5 mmol/L MgCl_2_·6H_2_O, 1 mmol/L EDTA, and 5 mmol/L KH_2_PO_4_, pH 7.4) and placed in a Clark electrode chamber (DW-1, UK). The reaction was initiated by the addition of 5 mmol/L succinate as substrate, and the rate of oxygen consumption was measured using O_2_ View software (https://www.hansatech-instruments.com, version O_2_ View 2.05, accessed on 19 August 2023). For COX activity determination, 0.1 mL of tissue homogenate (250 mmol/L sucrose, 5 mmol/L Tris, 1 mmol/L MgCl_2_·6H_2_O, 0.5 mmol/L EDTA, and 0.5 mg/mL BSA, pH 7.4) was mixed with 1.9 mL of COX reaction solution (7.5 mmol/L KH_2_PO_4_, 3.75 mmol/L ascorbic acid, 0.3 mmol/L N,N,N′,N′-tetramethyl-p-phenylenediamine dihydrochloride (TMPD), pH 7.4) and placed in the Clark electrode chamber. The reaction was initiated by the addition of 30 μmol/L cytochrome C as substrate, and the rate of oxygen consumption was recorded using O_2_ View software. Both S4R and COX activities were expressed as whole-organ consumption rates (μmol O_2_·min^−1^).

### 2.10. Determination of Crude Fat

The carcasses of mice were dried in an oven at 60 °C until a constant weight was reached. The dried carcasses were finely ground using a small grinder, and the weights of the empty filter paper and ground carcass samples were measured and recorded using an electronic balance to the nearest 0.001 g. A fixed amount (1 g) of carcass was weighed and wrapped in filter paper, and the total weight of the packages was recorded (to the nearest 0.001 g). The packages were then placed in a Soxhlet extractor and extracted with petroleum ether. At the end of the extraction procedure, the packages were removed and dried in an oven at 60 °C until the petroleum ether had completely evaporated, after which they were reweighed and recorded. The weight of fat extracted was calculated from the difference in the package mass before and after extraction, and the residual fat content in the carcass was subsequently calculated using following formula.

Residual carcass fat content (%) = (weight of filter paper + weight of sample − weight of filter paper pack after extraction)/weight of sample × 100% [47].

### 2.11. Software and Statistical Analysis

Data were analyzed using SPSS version 27.0 for Student’s *t*-tests, one-way analysis of variance (ANOVA), or repeated-measures ANOVA, as appropriate. Student’s *t*-test and repeated measures ANOVA were primarily applied to data from female mice, while repeated measures ANOVA was used for litter mass data. One-way ANOVA was mainly used for data from adult male offspring. Correlations between variables were assessed using Pearson’s correlation analysis. Data are expressed as mean ± standard error of the mean (SEM), with *p* < 0.05 considered statistically significant and *p* < 0.01 considered highly significant. Analysis of the intestinal flora of maternal and male offspring mice was primarily performed using the Meguiar’s Cloud platform (https://cloud.majorbio.com). Between-group differences in α-diversity were evaluated using the Wilcoxon rank-sum test, and principal coordinates analysis (PCoA) based on the Bray–Curtis distance was used to assess β-diversity. PCoA was further used to evaluate similarities in microbial community structure among samples, and one-way ANOVA was applied to test between-group differences in relative abundance at the phylum and genus levels.

## 3. Results

### 3.1. Body Mass and Food Intake in Lactating Mice and Their Offspring

**Figure 1:**
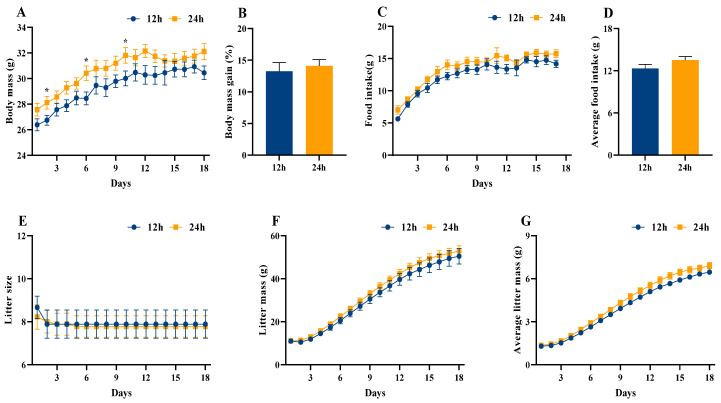
Effect of light exposure on body mass and food intake in lactating mice and their offspring. Body mass (**A**), body mass gain (**B**), food intake (**C**), and average food intake (**D**) in lactating female mice. Litter size (**E**), litter mass (**F**), and average litter mass (**G**) in their offspring. 12 h (12 h light exposure); 24 h (24 h light exposure). Data are expressed as mean ± SEM (*n* = 9). *, *p* < 0.05.

Maternal body mass and food intake increased significantly with advancing days of lactation (body mass: *F*_(17, 272)_ = 37.485, *p* < 0.001, Figure 1A; food intake: *F*_(16, 256)_ = 74.622, *p* < 0.001, Figure 1C). In contrast, neither continuous light exposure nor the interaction between days of lactation and continuous light exposure altered the temporal patterns of body mass and food intake (*p* > 0.05, Figure 1A,C). Consistently, no significant differences were observed between light treatments in maternal body mass gain, average food intake, and energy intake (*p* > 0.05, Figure 1B,D and Figure A3). Additionally, compared with the 12 h group, the 24 h group had a higher lung mass (*t*_16_ = −3.137, *p* = 0.006, Table A1). Detailed results are shown in Table A1.

Increasing days of lactation did not significantly affect litter size (*F*_(17, 272)_ = 0.623, *p* > 0.05, Figure 1E), and both litter mass and average litter mass increased markedly over time (litter mass: *F*_(17, 272)_ = 511.873, *p* < 0.001 Figure 1F; average litter mass: *F*_(17, 272)_ = 1564.022, *p* < 0.001, Figure 1G). In contrast, neither continuous light exposure nor the interaction between days of lactation and continuous light exposure significantly altered the trajectories of litter size and litter mass (*p* > 0.05, Figure 1E,F). However, the interaction between days of lactation and continuous light exposure significantly affected average litter mass (*F*_(17, 272)_ = 2.428, *p* = 0.002, Figure 1G). Among the tissues, only the large intestine weight (*F*_(2, 21)_ = 9.622, *p* = 0.001, Table A2) was significantly higher in the 12 h group than in the 24–12 h and 24 h groups. Detailed results are provided in Table A2.

### 3.2. Metabolism in Lactating Mice and Their Offspring

**Figure 2:**
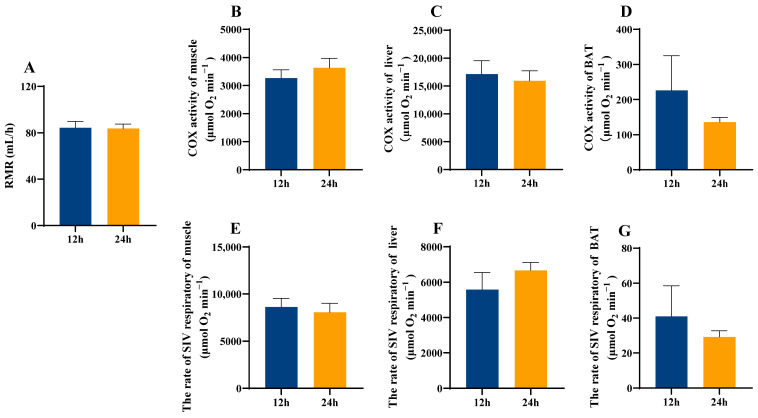
Effect of light exposure on metabolism in lactating mice. RMR (resting metabolic rate) (**A**), COX activity in muscle (**B**), liver (**C**), and BAT (**D**), and S4R levels in muscle (**E**), liver (**F**), and BAT (**G**) in lactating mice. 12 h (12 h light exposure); 24 h (24 h light exposure). Data are expressed as mean ± SEM (*n* = 9).

**Figure 3:**
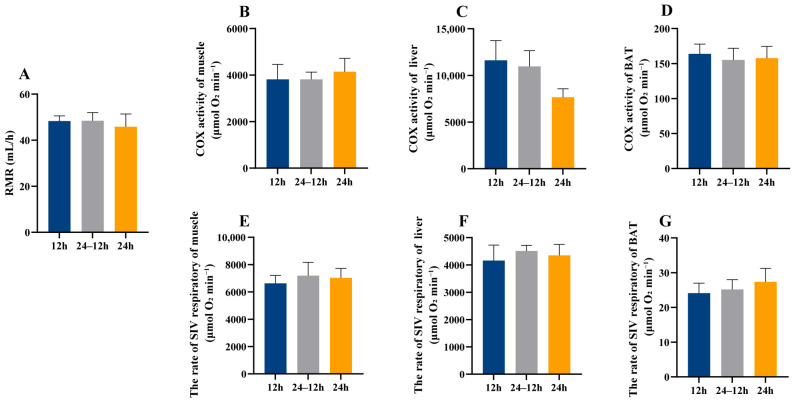
Effect of light exposure on metabolism in adult male offspring. RMR (resting metabolic rate) (**A**). COX activity in muscle (**B**), liver (**C**), and BAT (**D**). S4R levels in muscle (**E**), liver (**F**) and BAT (**G**). 12 h (12 h light exposure); 24–12 h (24 h light exposure during the lactation period, followed by a normal 12 h light:12 h dark cycle); 24 h (24 h light exposure). Data are expressed as mean ± SEM (*n* = 8).

Continuous light exposure had no significant effects on resting metabolic rate, muscle, liver, and BAT COX and S4R activities in lactating females and male offsprings (*p* > 0.05, Figure 2A–G and Figure 3A–G).

### 3.3. Immune and Antioxidant Capacities of Lactating Mice and Their Offspring

**Figure 4:**
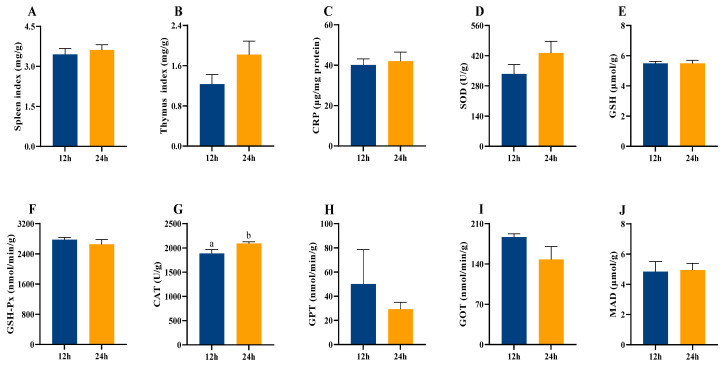
Effect of light exposure on immune and antioxidant capacities of lactating mice. Spleen index (**A**) and thymus index (**B**); CRP content (**C**), SOD (**D**), GSH (**E**), GSH-Px (**F**), CAT (**G**), GPT (**H**), GOT (**I**), and MDA (**J**) in liver. 12 h (12 h light exposure); 24 h (24 h light exposure). Data are expressed as mean ± SEM (*n* = 9). Different letters indicate significant differences between groups.

**Figure A1:**
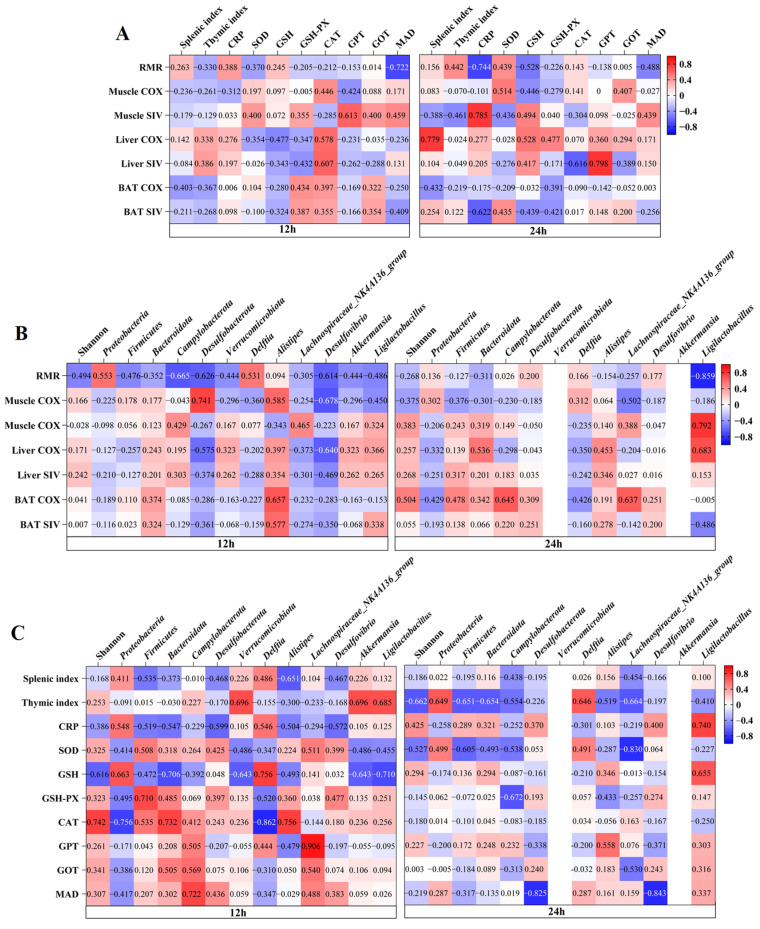
**Correlations among maternal metabolism, oxidative damage markers and gut microbiota in lactating mice.** Correlations between metabolism and oxidative damage (**A**). Correlations between metabolism and gut microbiota (**B**). Correlations between oxidative damage and gut microbiota (**C**). Red indicates positive correlation, while blue indicates negative correlation. The deeper the color, the stronger the correlation.

There were no significant differences in maternal spleen index, thymus index, and CRP (*p* > 0.05, Figure 4A–C) observed between the 12 h and 24 h groups of lactating mice. In contrast, CAT activity was significantly higher in the 24 h group than in the 12 h group (*t*_16_ = −2.255, *p =* 0.045, Figure 4G). Constant light exposure had no significant effects on the levels of SOD, GSH, GSH-Px, GPT, GOT, and MDA (*p* > 0.05, Figure 4D–F,H–J) in lactating females. Additionally, CAT activity was positively correlated with liver S4R level in the 12 h group but negatively correlated with liver S4R in the 24 h group (12 h: *r* = 0.607, *p* = 0.041, Figure A1A; 24 h: *r* = −0.616, *p* = 0.039, Figure A1A). This inconsistency was commonly observed when analyzing correlations between metabolism-related indices and the immune and oxidative damage parameters. Detailed results are provided in Figure A1A.

**Figure 5:**
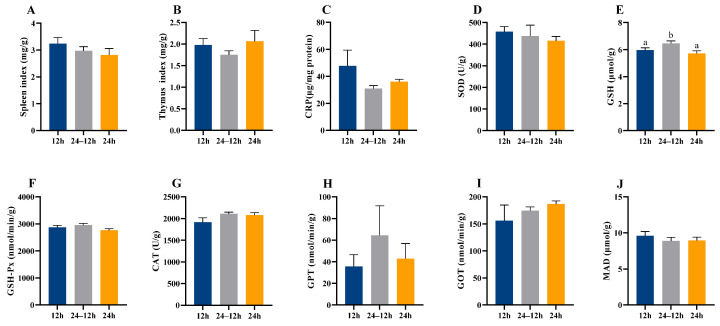
Effect of light exposure on immune and antioxidant capacities in adult male offspring. Spleen index (**A**); thymus index (**B**); liver CRP content (**C**); SOD activity (**D**); GSH content (**E**); GSH-Px activity (**F**); CAT activity (**G**); GPT activity (**H**); GOT activity (**I**); MDA content (**J**). 12 h (12 h light exposure); 24–12 h (24 h light exposure during the lactation period, followed by a normal 12 h light:12 h dark cycle); 24 h (24 h light exposure). Data are expressed as mean ± SEM (*n* = 8). Different letters indicate significant differences between groups.

**Figure A2:**
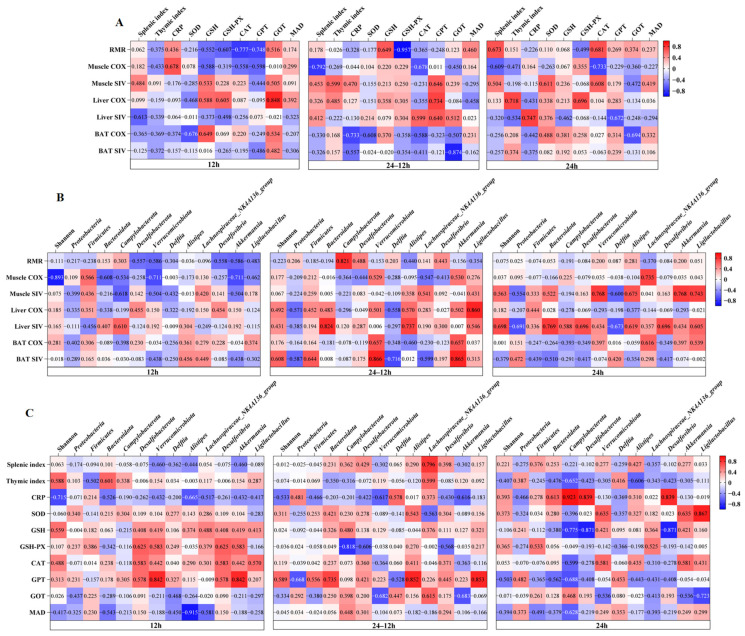
Correlations among maternal metabolism, oxidative damage markers and gut microbiota in male adult offspring. (**A**) Correlations between metabolism and oxidative damage. (**B**) Correlations between metabolism and gut microbiota. (**C**) Correlations between oxidative damage and gut microbiota. Red indicates positive correlation, while blue indicates negative correlation. The darker the color, the stronger the correlation.

**Figure A3:**
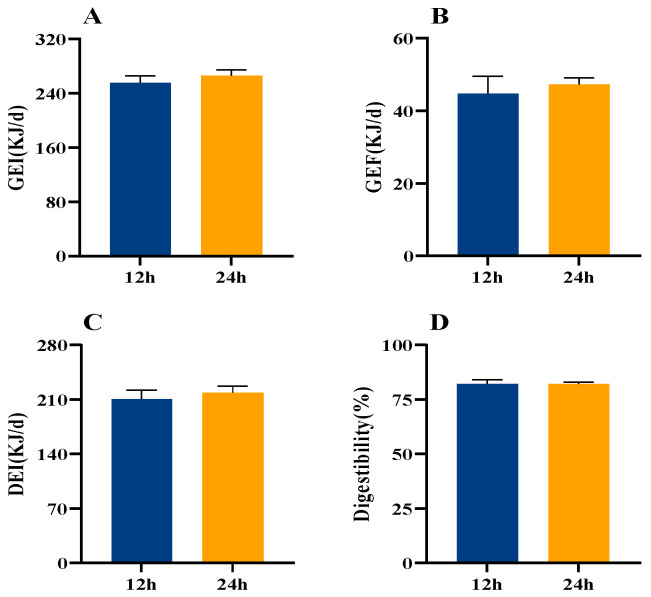
Effects of light exposure on energy intake in lactating mice. Gross energy of intake (GEI) (**A**), gross fecal energy (GEF) (**B**), digestive energy intake (DEI) (**C**), and digestibility (**D**) in lactating female mice. 12 h (12 h light exposure); 24 h (24 h light exposure). Data are expressed as mean ± SEM (*n* = 9).

In the case of male offspring, there were no significant differences in the spleen index, the thymus index, or the CRP content (*p* > 0.05, Figure 5A–C) among the 12 h, 24–12 h, and 24 h groups. In addition, the 24–12 h group showed a significant increase in GSH levels (*F*_(2, 21)_ = 4.425, *p* = 0.028; Figure 5E), compared with the other two groups. However, none of the three groups exhibited any significant changes in the levels of the SOD, GSH-Px, CAT, GPT, GOT, and MDA (*p* > 0.05, Figure 5D,F–J). Additionally, the correlation patterns of metabolism-related indices with immune and oxidative damage parameters differed significantly among the 12 h, 24–12 h, and 24 h groups. Detailed results are provided in Figure A2A.

### 3.4. The Gut Microbiota of Lactating Mice and Their Offspring

**Figure 6:**
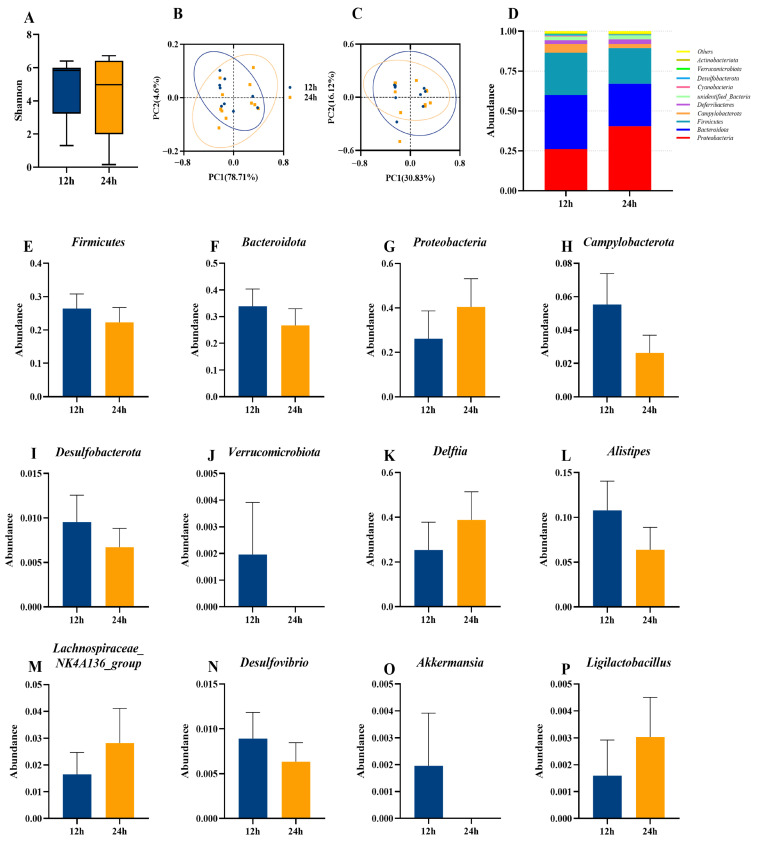
Effect of light exposure on the diversity and composition of the gut microbiota of lactating mice. Shannon index (α-diversity) of gut microbiota (**A**). Principal Coordinate Analysis (PCoA) plot based on weighted UniFrac distance (**B**) and unweighted UniFrac distance (**C**). Relative abundance of the top 10 taxa in the gut microbiota community at the phylum level (**D**). Relative abundance of *Firmicutes* (**E**), *Bacteroidota* (**F**), *Proteobacteria* (**G**), *Campylobacterota* (**H**), *Desulfobacterota* (**I**), and *Verrucomicrobiota* (**J**) at the phylum level. Relative abundance of *Delftia* (**K**), *Alistipes* (**L**), *Lachnospiraceae_NK4A136_group* (**M**), *Desulfovibrio* (**N**), *Akkermansia* (**O**), and *Ligilactobacillus* (**P**) at the genus level in the gut microbiota community. 12 h (12 h light exposure); 24 h (24 h light exposure). Data are expressed as mean ± SEM (*n* = 9).

In general, nocturnal light exposure had no significant effect on the gut microbiota composition of the lactating mice, as shown by a lack of significant difference in α-diversity (Shannon index) and β-diversity (PCoA) between the two groups (*p* > 0.05, Figure 6A–C). At the phylum level, *Proteobacteria*, *Bacteroidota*, *Firmicutes*, and *Campylobacterota* were the most abundant among the TOP 10 phyla (Figure 6D). However, we observed no significant differences between the 12 h and 24 h groups at the phylum and genus levels (*p* > 0.05, Figure 6E–P). Notably, *Verrucomicrobiota* and *Akkermansia* were not detected in the lactating mice in the 24 h light exposure group. The correlation patterns between gut microbial abundance and metabolism-related indices and between gut microbial abundance and immune and oxidative damage parameters differed significantly between the 12 h and 24 h groups (Figure A1B,C).

**Figure 7:**
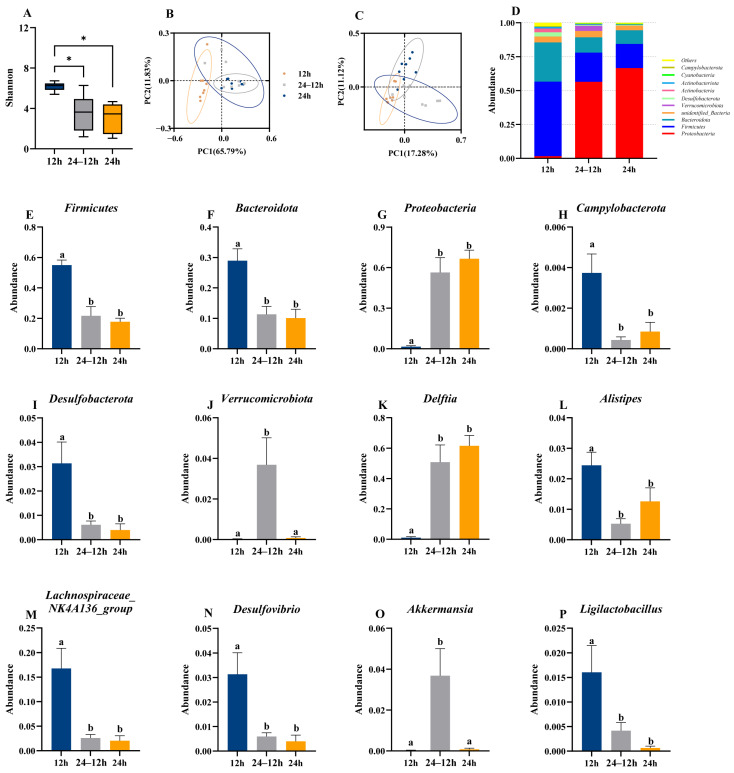
Effects of light exposure on the diversity and composition of the gut microbiota in male adult offspring. Shannon index (α-diversity) of gut microbiota (**A**). Principal Coordinate Analysis (PCoA) plot based on weighted UniFrac distance (**B**) and unweighted UniFrac distance (**C**). Relative abundance of the top 10 taxa in the gut microbiota community at the phylum level (**D**). Relative abundance of *Firmicutes* (**E**), *Bacteroidota* (**F**), *Proteobacteria* (**G**), *Campylobacterota* (**H**), *Desulfobacterota* (**I**), and *Verrucomicrobiota* (**J**) at the phylum level. Relative abundance of *Delftia* (**K**), *Alistipes* (**L**), *Lachnospiraceae_NK4A136_group* (**M**), *Desulfovibrio* (**N**), *Akkermansia* (**O**), and *Ligilactobacillus* (**P**) at the genus level in the gut microbiota community. 12 h (12 h light exposure); 24–12 h (24 h light exposure during the lactation period, followed by a normal 12 h light:12 h dark cycle); 24 h (24 h light exposure). *, *p* < 0.05. Data are expressed as mean ± SEM (*n* = 8). Different letters indicate significant differences between groups.

Male offspring α-diversity (Shannon index) was significantly lower in the 24–12 h and 24 h groups than in the 12 h group (*F*_(2, 21)_ = 12.612, *p* < 0.001, Figure 7A). β-diversity (PCoA) showed a clear separation between the 24–12 h and 24 h groups and the 12 h group (Figure 7B,C). At the phylum level, *Proteobacteria*, *Bacteroidota* and *Firmicutes* were also dominant among the top 10 phyla of gut microbiota in male offspring (Figure 7D). Among them, the abundance of *Proteobacteria* (*F*_(2, 21)_ = 22.786, *p* < 0.001, Figure 7G) was significantly higher in the 24–12 h and 24 h groups than in the 12 h group. Conversely, *Firmicutes* (*F*_(2, 21)_ = 23.348, *p* < 0.001, Figure 7E), *Bacteroidota* (*F*_(2, 21)_ = 11.094, *p* = 0.001, Figure 7F), *Campylobacterota* (*F*_(2, 21)_ = 7.235, *p* = 0.005, Figure 7H), and *Desulfobacterota* (*F*_(2, 21)_ = 8.162, *p* = 0.002, Figure 7I) were significantly lower in abundance in the 24–12 h and 24 h groups. In addition, *Verrucomicrobiota* (*F*_(2, 21)_ = 7.507, *p* = 0.003, Figure 7J) was significantly higher in the 24–12 h group than in the 12 h and 24 h groups. At the genus level, *Delftia* (*F*_(2, 21)_ = 17.683, *p* < 0.001, Figure 7K) abundance was significantly higher in the 24–12 h and 24 h groups than in the 12 h group. Conversely, *Lachnospiraceae_NK4A136_group* (*F*_(2, 21)_ = 11.222, *p* < 0.001, Figure 7M), *Alistipes* (*F*_(2, 21)_ = 6.82, *p* = 0.005, Figure 7L), *Desulfovibrio* (*F*_(2, 21)_ = 8.185, *p* = 0.002, Figure 7N), and *Ligilactobacillus* (*F*_(2, 21)_ = 5.991, *p* = 0.009, Figure 7P) abundances were significantly lower in the 24–12 h and the 24 h groups than in the 12 h group. In addition, *Akkermansia* (*F*_(2, 21)_ = 7.473, *p* = 0.004, Figure 7O) was significantly higher in the 24–12 h than in the other two groups. Nocturnal light exposure altered the composition of gut microbiota in male offspring, and though this effect appeared to originate during the lactation period, it was also partially dependent on the specific microbe. The correlation patterns between gut microbial abundance and metabolism-related indices and between gut microbial abundance and immune and oxidative damage parameters were comparable between the 24–12 h and 24 h groups but differed significantly from those in the 12 h group (Figure A2B,C).

## 4. Discussion

In this study, we investigated the effects of exposure to extreme ALAN on oxidative stress and gut microbiota composition in C57BL/6J mice during pregnancy, lactation, and postnatal development. Our results showed that continuous exposure significantly increased CAT activity in maternal mice and elevated GSH levels in the 24–12 h offspring. In parallel, offspring exposed to extreme ALAN exhibited a significant reduction in gut microbial α-diversity, accompanied by marked shifts in community composition, including decreases in *Firmicutes*, *Bacteroidota*, *Campylobacterota*, and *Desulfobacterota* abundance and an increase in Proteobacteria abundance. Additionally, the correlation patterns between gut microbial abundance and metabolism-related indices were comparable between the 24–12 h and 24 h groups but differed significantly from those in the 12 h group. However, the correlation patterns between gut microbial abundance and immune and oxidative damage parameters were comparable between the 12 h and 24–12 h groups but differed significantly from those in the 24 h group.

### 4.1. Effects of Nocturnal Artificial Light on Metabolism and Oxidative Stress on Maternal Mice

Photoperiod variation is known to influence physiological state, behavior, and redox balance in organisms [48]. Prolonged nocturnal artificial light exposure has been linked to obesity in humans [49]. However, our data revealed no significant changes in body mass, food intake, and metabolic rate of maternal mice subjected to continuous ALAN (Figure 1 and Figure 2), suggesting that exposure to continuous ALAN during pregnancy and lactation did not disrupt overall energy balance. Pregnancy and lactation represent peak periods of energy demand, during which energy homeostasis is tightly regulated, a key factor in maintaining body mass [50] This may reflect stage-specific physiological resilience during lactation. Nevertheless, continuous exposure to ALAN significantly increased the lung weight and dry mass of residual carcass. The lung is a metabolically active organ, playing a central role in respiration, while the residual carcass serves as a key site of metabolism [51,52]. In contrast, although there were no statistically significant differences in the masses of metabolically active tissues (liver, BAT, and muscle), as well as in COX activities and S4R levels across these tissues between the 12 h and 24 h groups, their trends differed substantially. These observations suggest that extreme ALAN may be associated with altered metabolic allocation in maternal mice.

In addition, continuous exposure to ALAN significantly increased CAT activity in maternal mice, and this increase was negatively correlated with hepatic S4R levels in the 24 h group, These results imply that the antioxidant system in 24 h group was compensatorily elevated to cope with extreme ALAN pressure, whereas the normal coupling between metabolism and antioxidant defense was disrupted, consistent with previous findings [53]. An upward trend in CRP levels was observed in the 24 h group, suggesting a mild inflammatory state under extreme ALAN exposure. Similar increases in plasma CRP levels have been reported in rodents exposed to ALAN [54]. SOD activity in the 24 h group also showed an approximate 29% increase relative to the control group (Figure 4D), indicating a trend toward enhanced antioxidant response. However, GPT and GOT activities did not differ significantly between groups (Figure 4H,I), indicating that extreme ALAN exposure during pregnancy and lactation did not induce overt hepatic injury.

### 4.2. Effects of Nocturnal Artificial Light on Metabolism and Oxidative Stress in Offspring Mice

Maternal physiological status and early-life experience are critically important for offspring development [53,55]. Consistent with maternal findings, nocturnal artificial light did not significantly affect body mass or metabolic rate in offspring mice, suggesting that gross energy balance remained stable during early development. This may be related to comparable maternal food intake across groups, resulting in similar nutritional provision to offspring. Previous studies have reported reduced antioxidant enzyme activity and elevated oxidative stress in rats exposed to constant light [56], whereas other species exhibit enhanced antioxidant capacity under altered photoperiods [57]. Our data showed that offspring mice that were initially continuously exposed to ALAN and subsequently maintained under a normal photoperiod after weaning exhibited significantly elevated hepatic GSH levels, relative to both continuously exposed and control groups. This increase may represent a compensatory antioxidant response following exposure to extreme ALAN early in life. One possible explanation is that exposure to extreme ALAN early in life induces lasting physiological adjustments, resulting in altered antioxidant defense regulation in adulthood. However, the continuous exposure to ALAN could lead to gradual adaptation in offspring mice, resulting in a relatively balanced response and regulation of antioxidant defense processes. These interpretations remain speculative and warrant further investigation. Additionally, we found that multiple antioxidant enzymes in the 24–12 h and 24 h groups exhibited parallel changes compared with 12 h group. This indicates that the effects of 24 h light exposure during early development are not fully abolished in adulthood. Similar findings have been reported in both mice and rats [55,58]. Mild fluctuations in antioxidant defense induced by continuous exposure in offspring also did not lead to significant changes in hepatic CRP levels. CRP is synthesized primarily in the liver and is widely recognized as a highly sensitive biomarker of inflammation and tissue damage [59]. Together, these results indicate that continuous light exposure in early life did not affect inflammatory activation in the offspring.

### 4.3. Effects of Nocturnal Artificial Light on the Gut Microbiota

Accumulating evidence indicates that exposure to ALAN can alter gut microbiota composition [60]. In maternal mice, extreme ALAN exposure resulted in the absence of *Verrucomicrobiota* and *Akkermansia*, indicating a potential inflammatory response. A previous study has shown that *Akkermansia* inversely correlates with body mass in rodents and humans [61]. The same study also demonstrates that *Akkermansia mucinphila*, a representative species, can exert anti-inflammatory effects and improve intestinal barrier function. Although no additional significant changes in maternal gut microbiota composition were detected, this pattern suggests that extreme ALAN exposure during pregnancy and lactation may elicit limited but targeted microbial responses, potentially buffered by the physiological regulatory capacity of maternal mice. Previous studies have shown that food intake in mice during peak lactation approximates half of their body mass [62]. Food intake is recognized as one of the most critical determinants of gut microbiota structure. The present study found no significant difference in food intake between the 12 h and 24 h maternal groups. Accordingly, we conclude that the influence of continuous ALAN on gut microbiota composition during pregnancy and lactation was largely masked by food intake.

At the offspring level, extreme ALAN exposure during early life led to pronounced gut microbiota alterations, inducing significant reductions in α-diversity, decreases in the abundance of the phyla *Firmicutes*, *Bacteroidota*, *Campylobacterota*, and *Desulfobacterota*, and reductions in the abundance of the genera *Alistipes, Lachnospiraceae_NK4A136_group*, *Desulfovibrio*, and *Ligilactobacillus*. Conversely, *Proteobacteria* and *Delftia* were significantly enriched. Notably, these microbial alterations were not reversed under the photoperiod normalization conditions tested, indicating persistent effects of early-life exposure to extreme ALAN on gut microbial structure. There is evidence indicating that young mice prefer much lower illumination than adults [63]. Therefore, we speculate that the offspring exhibit a strong response to early-life continuous light exposure. Previous studies have also associated enrichment of *Campylobacterota*, *Desulfobacterota*, *Proteobacteria*, *Delftia*, *Alistipes*, and *Desulfovibrio* with inflammatory processes [64,65,66,67,68], whereas *Ligilactobacillus* species and *Lachnospiraceae taxa* are generally considered beneficial as these bacteria have been shown to exert anti-inflammatory effects by suppressing cytokine production and restoring immune homeostasis [69,70]. However, these microbiota exhibited inconsistent patterns of change, suggesting an overall dynamic profile characterized by distinct responses among different phyla to ALAN exposure. Such microbial dysbiosis fundamentally compromises intestinal mucosal barrier integrity, thereby precipitating chronic systemic inflammation [64,65,66]. Regrettably, intestinal barrier integrity and inflammatory cytokines were not directly measured in this study; however, the observed microbial shifts are partially consistent with a microbiota profile associated with inflammatory susceptibility [64,68]. Notably, *Verrucomicrobiota* and *Akkermansia* were significantly increased in the 24–12 h group, suggesting that early-life exposure to extreme ALAN may have long-lasting effects on microbial colonization trajectories, even after returning to normal light conditions. Although previous studies have demonstrated that *Akkermansia* can exert anti-inflammatory effects [61], the specific reason why the 24–12 h group showed significantly higher levels than the other two groups remains unclear. Further correlation analysis between oxidative stress and gut microbiota revealed that *Akkermansia* abundance was positively correlated with CAT activity in the 12 h and 24 h groups but negatively correlated with CAT activity in the 24–12 h group. This discrepancy may be attributed to fluctuating light conditions, and warrants further investigation.

## 5. Conclusions

In summary, exposure to artificial light at night altered antioxidant defense status and gut microbiota composition in mice, with distinct effects observed between maternal and offspring generations. In maternal mice, the effects of short-term exposure appeared to be partially counterbalanced by antioxidant regulation and limited microbial changes. In contrast, offspring subjected to extreme ALAN exhibited persistent alterations in oxidative markers and gut microbiota composition that were not reversed under the experimental photoperiod recovery conditions tested. These results demonstrate that extreme ALAN exposure is associated with measurable changes in oxidative status and gut microbiota structure.

Despite these meaningful results, several limitations should also be acknowledged. All experiments were conducted under controlled laboratory conditions, and gut barrier integrity, inflammatory cytokines, circadian rhythm-related genes, behavior, and breast milk composition were not directly assessed. Among them, nutrients and bioactive components in breast milk may mediate the effects of maternal light exposure on offspring development and gut microbiota colonization. Further studies integrating breast milk composition, behavioral rhythm analyses, sex-specific differences in offspring, long-term recovery paradigms, and alternative photoperiod recovery strategies will be essential to fully elucidate the physiological and ecological consequences of extreme ALAN exposure. Nevertheless, the findings of the present study do highlight the complexity of extreme ALAN-induced biological effects and underscore the need for careful evaluation of ALAN as an environmental factor influencing mammalian health and gut microbiota across generations. Importantly, these findings provide an experimental basis for future exploration of the physiological consequences of ALAN.

## Data Availability

The original contributions presented in this study are included in the article. Further inquiries can be directed to the corresponding author.

## Abbreviations

The following abbreviations are used in this manuscript:

ALAN	Artificial light at night
ANOVA	Analysis of variance
BAT	Brown adipose tissue
CAT	Catalase
COX	Cytochrome c oxidase
CRP	C-reactive protein
DEI	Digestible energy intake
DMR	Daily metabolic rate
EDTA	Ethylenediaminetetraacetic acid
GEF	Gross energy of fecal
GEI	Gross energy intake
GOT	Aspartate aminotransferase
GPT	Alanine aminotransferase
GSH	Reduced glutathione
GSH-Px	Glutathione peroxidase
IOI	Immune organ index
MDA	Malondialdehyde
RMR	Resting metabolic rate
ROS	Reactive oxygen species
S4R	State IV respiration
SOD	Superoxide dismutase
TMPD	N,N,N′,N′-tetramethyl-p-phenylenediamine

## Appendix A

**Table A1:** Effects of light exposure on weight of organs in lactating mice.

Unit (g)	12 h	24 h	*P*_12h vs. 24h_
BAT	0.138 ± 0.063	0.080 ± 0.006	0.437
Liver	1.973 ± 0.248	2.122 ± 0.070	0.649
Heart	0.274 ± 0.020	0.262 ± 0.016	0.476
Lung	0.172 ± 0.008 ^b^	0.199 ± 0.008 ^a^	0.006
Small intestine	1.365 ± 0.161	1.577 ± 0.093	0.173
Large intestine	0.270 ± 0.016	0.244 ± 0.014	0.182
Stomach	0.304 ± 0.026	0.367 ± 0.020	0.085
Kidney	0.365 ± 0.009	0.376 ± 0.011	0.551
Subcutaneous fat	0.898 ± 0.069	0.891 ± 0.072	0.522
Mesenteric fat	0.450 ± 0.022	0.466 ± 0.032	0.864
Abdominal fat	0.029 ± 0.003	0.030 ± 0.003	0.839
Caecum	0.274 ± 0.017	0.307 ± 0.016	0.395
Dry mass of residual carcass	5.265 ± 0.093 ^b^	5.601 ± 0.100 ^a^	0.026
Body fat content%	16.421 ± 1.323	18.764 ± 1.642	0.283
Body fat mass	0.866 ± 0.076	1.057 ± 0.104	0.160

Data are means ± s.e.m. Different letters indicate significant differences between groups.

**Table A2:** Effects of light exposure on weight of organs in male adult offspring.

Unit (g)	12 h	24 h	24–12 h	*P*_12h vs_ _. 24h vs__. 24–12h_
BAT	0.088 ± 0.004	0.080 ± 0.006	0.085 ± 0.005	0.554
Liver	1.367 ± 0.063	1.265 ± 0.028	1.366 ± 0.026	0.173
Heart	0.171 ± 0.012	0.158 ± 0.008	0.148 ± 0.002	0.180
Lung	0.160 ± 0.005	0.162 ± 0.008	0.151 ± 0.005	0.416
Small intestine	0.983 ± 0.038	1.029 ± 0.050	1.069 ± 0.048	0.426
Large intestine	0.296 ± 0.024 ^a^	0.214 ± 0.011 ^b^	0.195 ± 0.014 ^b^	0.001
Stomach	0.296 ± 0.033	0.284 ± 0.017	0.356 ± 0.023	0.131
Kidney	0.399 ± 0.069	0.453 ± 0.021	0.449 ± 0.026	0.219
Subcutaneous fat	0.138 ± 0.019	0.144 ± 0.023	0.193 ± 0.020	0.117
Mesenteric fat	0.171 ± 0.011	0.154 ± 0.003	0.146 ± 0.008	0.634
Abdominal fat	0.372 ± 0.022	0.378 ± 0.008	0.344 ± 0.007	0.148
Caecum	0.167 ± 0.012	0.139 ± 0.011	0.143 ± 0.007	0.094

Data are means ± s.e.m. Different letters indicate significant differences between groups.
